# Centering the Voices of Dual-Role Caregivers Through Poetic Inquiry

**DOI:** 10.1093/geroni/igaf122.3555

**Published:** 2025-12-31

**Authors:** Charlotte Weiss, Karen Hirschman, Mary Naylor

**Affiliations:** University of Pennsylvania, Philadelphia, Pennsylvania, United States; University of Pennsylvania, Philadelphia, Pennsylvania, United States; University of Pennsylvania, Philadelphia, Pennsylvania, United States

## Abstract

Dual-role caregivers, those who are paid caregivers at work and informal caregivers at home, provide invaluable services to society, and their stories should be heard and valued. In our primary study, we interviewed eight women of color employed at a long-term care (LTC) facility in Pennsylvania, all of whom also served as informal caregivers of family members in their personal lives. These interviews explored the complexities of balancing professional caregiving responsibilities with familial caregiving demands. As a secondary analysis, we used a qualitative, arts-based research method of poetic inquiry, known as I-Poems, to enhance the visibility and representation of dual-role caregivers. Poetic inquiry, as a postmodern and feminist-informed research method, is particularly well-suited to study and represent caregivers’ lives. I-Poems were created by lifting the participants’ statements representing or speaking of self from an interview text and positioning them into lines of a poem that were then constructed into poetic stanzas. By separating the “I-voice,” the participants’ sense of agency and self is repositioned to the forefront of the caregiver story. We will present multiple I-Poems to illustrate the contextual intersections of caregiving at work and at home. These poetic representations invite the audience to feel, rather than simply understand, the multidimensional and embodied realities of dual-role caregiving. These participant-voiced research poems contribute to new knowledge and foster humanization of caregivers who navigate care responsibilities across both professional and personal spheres.

